# Supplementary material for: The Healthy Eating Plate Advice for Migraine Prevention: An Interventional Study
Source: Nutrients. 2020 May 28;12(6):1579. doi: 10.3390/nu12061579 (PMC7352548; doi:10.3390/nu12061579)
Supplement: Supplementary file 1 [file nutrients-12-01579-s001.pdf]

Supplementary table S1.

Food groups	Food Items
Fresh fruits	All kind of fresh fruits
Vegetables	All kind of vegetables (excluding potatoes)
Milk and Diary	Milk, Yogurt, Mozzarella
Whole grain cereals	Whole grain pasta, whole grain rice, spelt, barley, whole grain bread
Refined cereals-potatoes	Refined grain Pasta, Refined grain Rice, Bread
High carb breakfast-snacks	Confectionary sweet products, croissants, refined grain biscuits,
	refined grain breakfast cereals, cakes, French fries, fried rice balls
Whole grain breakfast	Whole grain breakfast cereals, whole-grain biscuits
Vegetable oils	Olive oil, Corn Oil, Seed Oil
Animal fat and margarine	Butter, lard, margarine
Cheese	Soft cheese and seasoned cheese (goat and cow milk)
Egg	Egg
Legumes	Different kind of beans, peas, lentil
White meat	Chicken, turkey
Red and processed meat	Beef, lamb, pork, sausage, ham, salami, and any processed meat
Fish	Fresh or Frozen fish (or mussel)
Sweetened beverage	Soft drinks, confectionary juices
Nuts	walnuts, chestnuts, peanuts, hazelnuts, pistachio, almonds, seeds
Alcohol	Wine, beer, spirits
